# Editorial: Nutritional assessment tools for identification and monitoring of malnutrition in patients with chronic disease, volume II

**DOI:** 10.3389/fnut.2023.1211518

**Published:** 2023-07-18

**Authors:** Eloisa Colin-Ramírez, Lilia Castillo-Martínez

**Affiliations:** ^1^School of Sports Science, Universidad Anáhuac México, Huixquilucan de Degollado, State of Mexico, Mexico; ^2^Servicio de Nutriología Clínica, Instituto Nacional de Ciencias Médicas y Nutrición Salvador Zubirán, Mexico City, Mexico

**Keywords:** chronic disease-related malnutrition, assessment, sarcopenia, nutritional status, chronic disease, tools

The European Society for Clinical Nutrition and Metabolism (ESPEN) recognized three different types of malnutrition (or undernutrition), which includes disease-related malnutrition (DRM) with and without inflammation, and malnutrition/undernutrition without disease (1). DRM is highly prevalent among hospitalized patients, with a prevalence rate ranging between 20 and 50% (2). The clinical and economic burden of DRM points out the need for timely identification and treatment of this clinical condition; however, inpatients are not often assessed for DRM, in part due to the lack of standardized criteria for its diagnosis (3–5). Of critical importance is that some common biomarkers, such as serum concentrations of visceral proteins used to assess malnutrition, may not be valid to assess or monitor malnutrition in the context of DRM with inflammation, since they may be affected by the underlying disease-related inflammation process (6).

The Global Leader Initiative on Malnutrition (GLIM) proposed criteria for the diagnosis of malnutrition, including unintentional weight loss, low BMI, and decreased muscle mass as phenotypic criteria, and impaired food intake or assimilation and burden of disease/inflammation as etiologic criteria. To diagnose malnutrition, at least one phenotypic, and one etiological criterion are required; however, criteria for determining inflammation as etiology are not provided, authors only mention that C-reactive protein (CRP), albumin, or pre-albumin could be proxy measures (7). DRM with inflammation according to ESPEN is a catabolic condition triggered by a disease-specific inflammatory response, including anorexia and tissue breakdown (1). Validation studies for the GLIM criteria are still needed to test its validity for the diagnosis of malnutrition in diverse patient populations. Also, sarcopenia should not be considered equivalent to malnutrition but a part of the definition to include skeletal muscle mass and function indicators.

This Research Topic is a second edition of the Nutritional Assessment Tools for Identification and Monitoring of Malnutrition in Patients with Chronic Disease, which addresses the current and novel nutritional assessment tools for the identification and monitoring of malnutrition in patients with chronic disease. In volume 1, a total of 12 articles were included, covering different aspects of malnutrition, such as sarcopenia, its prognosis value, predictor factors, and potential therapeutic strategies, among other relevant topics. Due to the high interest expressed in this Research Topic and the number of meaningful contributions received, volume 2 was released.

In this second edition, 13 articles were published. Eight out of 13 studies provided evidence of the clinical relevance and prognosis value of diverse indexes to screen for nutritional risk and assess malnutrition in different patient populations. Kang et al. showed that the mini nutritional assessment (MNA) screening tool has a better performance to predict various negative outcomes, including 3-month all-cause mortality and geriatric syndrome, compared to serum albumin, one of the biomarkers most commonly used for nutritional assessment, in hospitalized older patients. Another study on the geriatric population conducted by Peng et al. demonstrated that a high risk of malnutrition status identified by the geriatric nutrition risk index (GNRI) was able to predict poor prognosis in elderly patients in the intensive care unit (ICU) setting. The GNRI was also tested along with other nutritional and inflammatory markers for predicting overall survival in early-onset colorectal cancer in a study carried out by Xiang et al.. Authors found that among all nutritional and inflammatory indicators studied, the systemic immune inflammation index (SII) and the GNRI had higher prognostic values, and both were correlated with tumor stage. The prognostic nutritional index (PNI), an indicator of nutritional status and systemic inflammation, was tested for the first time in decompensated liver cirrhosis by Xie et al.. They showed an association between decreased PNI and increased risk of death, suggesting that PNI may be a prognostic marker in this patient population. Also, PNI was identified to be associated with the decision-making on the choice of renal replacement therapy (RRT) modality in adults with advanced chronic kidney disease (ACKD) in a study by Álvarez-García et al.. PNI score was significantly lower in patients who chose home-based RRT (18.8%) compared with in-center RRT (81.2%). However, in the multivariate binary regression analysis, it was not independently associated with the free decision-making to choose in-center and home-based RRT modality but were age, Charlson comorbidity index, follow-up at ACKD unit >6 months, and serum albumin. Chen D. et al. studied the prognostic performance of the Nutritional Risk Screening 2002 (NRS 2002), the Nutrition Risk in Critically Ill (NUTRIC) score, and modified Nutrition Risk in Critically Ill (mNUTRIC) nutritional screening tools that for the first time in patients with severe acute pancreatitis. The three studied scores were predictors for mortality at 28- and 90-days; however, the NUTRIC and mNUTRIC showed better predictive ability in this patient population. In China, the GLIM criteria were modified by removing the muscle-related indicators since these are not based on Chinese population standards. Guo et al. examined the effects of the GLIM-China on the diagnosis of malnutrition in patients with hematopoietic stem cell transplants. Authors concluded that a large proportion of patients with reduced muscle mass indicators will be missed for the diagnosis of malnutrition by using the GLIM-China, highlighting the relevance of muscle mass indicators for the diagnosis of malnutrition.

Two studies addressed the importance of body composition parameters in nutritional status assessment and its role in predicting the risk of clinical conditions. A mini-review by Wu et al. evaluated the role of bioelectrical impedance analysis-derived phase angle as a predictive marker for sarcopenia in patients with cancer and non-cancer diseases, suggesting that phase angle is an emerging and reliable predictor of sarcopenia in patients with different types of cancer; however, its association with non-cancer conditions is less clear. Also, further investigation is needed to determine cutoff values to screen for pre-sarcopenia and sarcopenia. Additionally, Kuang et al. conducted a study to assess the contribution of body composition fat mass (FM) and lean body mass (LBM) to non-alcoholic fatty liver disease (NAFLD), demonstrating higher LBM is associated with a lower risk of NAFLD and higher FM increasing the risk of this condition, particularly in women. Undoubtedly, body composition analysis represents a key aspect of nutritional status assessment; however, any nutritional assessment needs to guide a decision-making process to manage malnutrition when identified. In this sense, in a retrospective study of patients with Crohn's disease, Jiang et al. confirmed that pre-operative nutritional status correlates with post-operative outcomes and that enteral nutrition was associated with an improvement in nutritional parameters and a reduced rate of postoperative complications in patients with Crohn's disease undergoing surgery.

Evidence on the usefulness of other technologies such as 3D facial image recognition was also provided in this Research Topic. Chen M. et al. reported that the facial temporal region and periorbital depression indicators extracted by 3D image recognition technology were associated with the phenotype of malnutrition-related muscle and fat loss in patients with cancer, providing an alternative for a clinical auxiliary tool for malnutrition screening and assessing phenotypic indicators of malnutrition.

Finally, Domenech-Briz et al. contributed with a systematic review of 14 the importance of nutritional assessment tools in critically ill patients pointed out the benefits of screening or assessing malnutrition for predicting mortality risk and early initiation of nutritional therapy, reducing the number of complications and length of stay related to malnutrition or adjusting energy requirements. Authors concluded that among the studied tools, the most widely used and effective were the modified Nutrition Risk in the Critically Ill (mNUTRIC) score, the Nutrition Risk Screening 2002, and the Subjective Global Assessment, either independently or in combination with each other.

In conclusion, further studies are needed to demonstrate that in addition to the identification of DRM, with above mentioned nutritional assessment tools, nutritional and exercise interventions are justified, and changes in nutrition outcomes could be detected after these interventions. It is crucial to include a decision-making process to guide the management when malnutrition is present.

## Author contributions

EC-R wrote the introduction and central part with comments on the cited papers and references. LC-M wrote the conclusion and reviewed/edited the introduction and central part. All authors contributed to the article and approved the submitted version.
